# Two-Experiment Examination of Habitual and Manipulated Foot Placement Angles on the Kinetics, Kinematics, and Muscle Forces of the Barbell Back Squat in Male Lifters

**DOI:** 10.3390/s22186999

**Published:** 2022-09-15

**Authors:** Jonathan Sinclair, Paul John Taylor, Gareth Shadwell, Mark Stone, Nicole Booth, Bryan Jones, Sam Finlay, Ashraf Mohamed Ali, Bobbie Butters, Ian Bentley, Christopher James Edmundson

**Affiliations:** 1 Research Centre for Applied Sport, Physical Activity and Performance, School of Sport & Health Sciences, Faculty of Allied Health and Wellbeing, University of Central Lancashire, Preston PR1 2RA, UK; 2 School of Psychology & Computer Sciences, Faculty of Science & Technology, University of Central Lancashire, Preston PR1 2RA, UK; 3 Wigan Warriors RLFC, Wigan WN5 0UH, UK

**Keywords:** biomechanics, squat, kinetics, kinematics, muscle forces

## Abstract

This two-experiment study aimed to examine the effects of different habitual foot placement angles and also the effects of manipulating the foot placement angle on the kinetics, three-dimensional kinematics and muscle forces of the squat. In experiment 1, seventy lifters completed squats at 70% of their one repetition maximum using a self-preferred placement angle. They were separated based on their habitual foot angle into three groups HIGH, MEDIUM and LOW. In experiment 2, twenty lifters performed squats using the same relative mass in four different foot placement angle conditions (0°, 21°, 42° and control). Three-dimensional kinematics were measured using an eight-camera motion analysis system, ground reaction forces (GRF) using a force platform, and muscle forces using musculoskeletal modelling techniques. In experiment 1, the impulse of the medial GRF, in the descent and ascent phases, was significantly greater in the HIGH group compared to LOW, and in experiment 2 statistically greater in the 42° compared to the 21°, 0° and control conditions. Experiment 2 showed that the control condition statistically increased quadriceps muscle forces in relation to 0°, whereas the 0° condition significantly enhanced gluteus maximus, gastrocnemius and soleus forces compared to control. In experiment 1, patellofemoral joint stress was significantly greater in the HIGH group compared to LOW, and in experiment 2, patellar and patellofemoral loading were statistically greater in the control compared to the 42°, 21°, 0° and control conditions. Owing to the greater medial GRF’s, increased foot placement angles may improve physical preparedness for sprint performance and rapid changes of direction. Reducing the foot angle may attenuate the biomechanical mechanisms linked to the aetiology of knee pathologies and to promote gluteus maximus, gastrocnemius and soleus muscular development. As such, though there does not appear to be an optimal foot placement angle, the observations from this study can be utilised by both strength and conditioning and sports therapy practitioners seeking to maximise training and rehabilitative adaptations.

## 1. Introduction

The barbell back squat is one of the most frequently adopted resistance exercises for development purposes in athletic disciplines that necessitate high levels of strength and power [1]. Because of its ability to recruit the quadriceps, hamstrings, tibialis anterior, gastrocnemius, soleus and lumbar muscles [2], and its function as a closed kinetic chain exercise, it is also commonly utilised in rehabilitation settings [3].

This exercise has received considerable attention in strength and conditioning literature due to its functionality and applicability to such a wide array of athletic environments. Due to its adaptability, multiple variations and technique manipulations can be made to the barbell back squat to mediate different mechanical outcomes [4]. Commonly explored adaptations include squat depth, stance width and foot placement angle [5]. The different types of executions may influence the biomechanics of the squat; thus, specific variations in squat techniques may be optimally modified in order to better achieve specific training stimuli and rehabilitative outcomes. 

There has been a plethora of peer-reviewed analyses concerning the effects of both squat depth and stance width on the biomechanics of the squat. Chan et al. [6] explored the effects of full and parallel depth squats and showed that the peak internal knee extension moment was increased significantly in the full depth condition. Caterisano et al. [7] examined the effects of partial, parallel, and full-depth squats on the relative contributions of lower extremity muscle potentials using electromyography. Their observations showed that relative gluteus maximus activation during the concentric phase of the squat increased linearly with squat depth. In terms of stance width, McCaw and Melrose, [8] examined widths of 75 and 140% shoulder distance on lower extremity muscle activation during the back squat with low and high loads. Muscle activity in the gluteus maximus and adductor longus muscles across both loads were significantly larger in the wide stance condition. Escamilla et al. [4] examined narrow, medium and wide groups based on their self-selected stance width. Their observations showed that the hip was in a significantly more flexed position and the knee extensor and ankle plantarflexor moments were significantly greater in the wide and medium conditions compared to narrow. Paoli et al. [2] showed that gluteus maximus activation was significantly greater in the wide squat condition. Lahti et al. [9] examined wide (1.5× greater trochanter width) and narrow (1.0 greater trochanter width) barbell back squats. Their results showed firstly, that the knee flexion angle and the angle of the resultant ground reaction force (GRF) vector were larger in the narrow and the hip-to-knee joint extension moment ratio was significantly greater in the wide stance condition.

However, the foot placement angle has received a paucity of research attention, with only one published output having examined the effects of this phenomenon on the biomechanics of the back squat. Using a repeated measures study design, Lorenzetti et al. [5] explored the effects of manipulating the foot placement angles to 0°, 21° and 42° on knee displacement, range of motion at the hip and knee joints, and joint moments at the hip, knee, and lower back. Their observations showed that both lateral knee displacement and hip abduction range of motion were significantly greater in the 21° and 42° foot angle conditions compared to 0°. However, Lorenzetti et al. [5] did not examine the influence of manipulating the foot placement angle on GRF’s or muscle kinetics, and there has not yet been any consideration within the scientific literature of the effects of habitual foot placement angles on the biomechanics of the squat. Therefore, controversy exists regarding the most effective foot placement angle position during the barbell back squat.

Currently, there has yet to be any investigation that has concurrently examined the influence of foot placement angle on the kinetics, three-dimensional kinematics and muscle forces during the back squat. Therefore, such an investigation may provide further insight regarding the effects of technique manipulations on biomechanical outcomes during the barbell back squat that may be important for strength and conditioning coaches and sports therapists seeking to promote distinct training stimuli and rehabilitative adaptations. As such, the aims of the current investigation are twofold. Firstly, experiment 1 used a between-participants’ design to comparatively examine the effects of different habitual foot placement angles on kinetics, three-dimensional kinematics and muscle forces during the squat. Secondly, using a repeated measures study design, experiment 2 explored the effects of manipulating the foot placement angle on the same biomechanical parameters. 

## 2. Materials and Methods

### 2.1. Ethical Approval

The procedures used for this investigation were approved by the ethical committee of the University of Central Lancashire (Reference = 458).

### 2.2. Experiment 1

#### 2.2.1. Participants

An a priori sample size calculation for independent group comparisons was undertaken with expected unequal group sizes, using the formulae outlined by Rosner [10]. With the squat representing a fundamental powerlifting exercise and with previous analyses showing in field and court sport athletes that peak power output is most predictive of elite athletes’ performance [11], it was determined that the peak power during the squat was the most appropriate measure to serve as the primary dependent variable. Currently, a minimum important difference (MID) for this parameter does not exist within the scientific literature, therefore using data from our previous work [12], in accordance with Sinclair et al. [13], the MID was calculated using a distribution-based approach to detect a difference of 1.95 W/kg between groups. It was determined that in order to achieve α = 5% and β = 0.80, a total of 70 participants would be required. A male-only cohort of lifters (age: 29.25 ± 5.40 years, stature: 177.25 ± 5.76 cm, mass: 81.14 ± 9.88 kg and 1RM back squat: 130.45 ± 22.79 kg) volunteered to take part in the current study. Participants were all practiced in the high bar back squat with a minimum of 2 years of training experience in this lift. All were free from musculoskeletal pathology at the time of data collection and provided written informed consent.

#### 2.2.2. Procedure

Three-dimensional kinematics were captured using an eight-camera motion analysis system (Qualisys Medical AB, Goteburg, Sweden) which sampled at 250 Hz. In addition, to capture GRF data, piezoelectric force plates (Kistler, Kistler Instruments Ltd., Alton, Hampshire, UK) were adopted, which collected data at 1000 Hz. Kinematics and GRF information were synchronously collected using an analogue-to-digital interface board.

**Figure 1:**
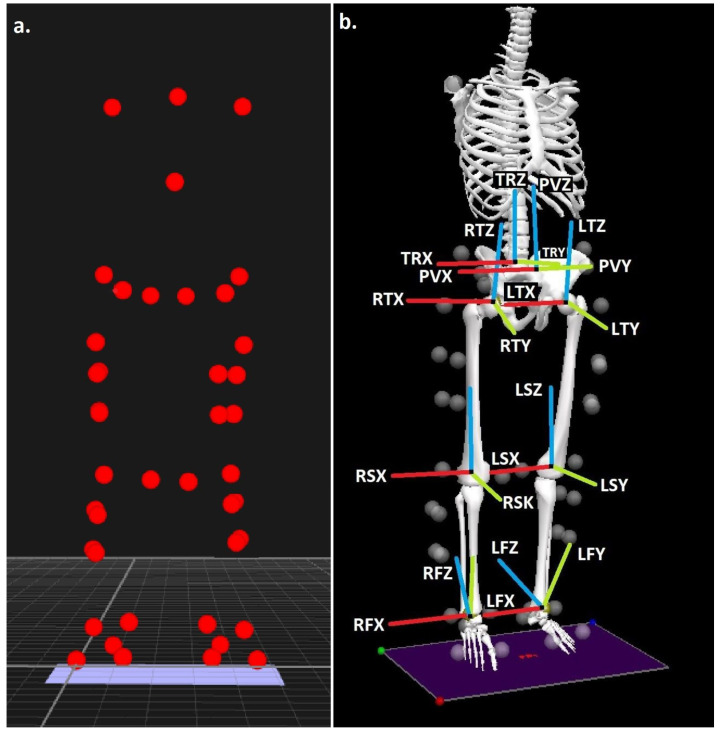
(**a**) Experimental marker locations and (**b**) trunk, pelvis, thigh, shank, and foot segments, with segment co-ordinate system axes (R = right and L = left), (TR = trunk, P = pelvis, T = thigh, S = shank, and F = foot), (X = sagittal, Y = coronal, and Z = transverse planes).

Body segments were modelled in 6 degrees of freedom using the calibrated anatomical systems technique [14], using a marker configuration utilised previously to quantify the biomechanics of the squat [12] (Figure 1a). The anatomical frames of the torso, pelvis, thighs, shanks and feet were delineated via the retroreflective marker. Carbon-fiber tracking clusters comprising four non-linear retroreflective markers were positioned onto the thigh and shank segments. In addition to these, the foot segments were tracked via the calcaneus, first metatarsal and fifth metatarsal, the pelvic segment using the posterior superior iliac and anterior superior iliac spine markers, as well as the torso via 7th cervical vertebrae, 12th thoracic vertebrae and xiphoid processes. Finally, a further two markers were positioned at either end of the bar allowing the bar to be delineated as a segment, permitting bar kinematics to be explored. The centre of the ankle and knee joints were delineated as the mid-point between the malleoli and femoral epicondyle markers [15,16], whereas the hip joint centre was obtained using the positions of the ASIS markers [17].

Static calibration trials (not normalised to static trial posture) were obtained with the participant in the anatomical position in order for the positions of the anatomical markers to be referenced in relation to the tracking clusters/markers. A static trial was conducted with the participant in the anatomical position in order for the anatomical positions to be referenced in relation to the tracking markers, following which those not required for dynamic data were removed. The Z (transverse) axis was oriented vertically from the distal segment end to the proximal segment end. The Y (coronal) axis was oriented in the segment from posterior to anterior. Finally, the X (sagittal) axis orientation was determined using the right-hand rule and was oriented from medial to lateral (Figure 1b).

#### 2.2.3. Squat Protocol

For data collection, all participants presented to the laboratory at least 48 h after their previous lower-body resistance training session. Before the measured squats were initiated, a general warm-up was completed, including a pulse raiser, dynamic stretches and potentiation exercises, followed by squat warm-up sets with 30 and 50% of 1RM [12]. Participants completed five continuous high bar back squat repetitions at 70% of their 1 repetition maximum (1RM) with their normal squat technique, using standardised experimental footwear (Adidas Powerlift, 3.0). Foot placement angles were calculated as the transverse plane angle of the foot segment relative to the laboratory co-ordinate system. A load of 70% of 1RM was selected in accordance with Sinclair et al. [18] and was deemed to be representative of a typical training load, whilst still maintaining the levels of repeatability necessary obtain a representative data set. In accordance with the National Strength and Conditioning Association (NSCA) guidelines, lifters were instructed to descend in a controlled manner, keep both feet flat on the floor, preserve proper breath control and maintain a constant/stable pattern of motion for each repetition. Each participant was examined visually by an NSCA certified strength and conditioning specialist.

#### 2.2.4. Processing

Marker trajectories were digitised using Qualisys Track Manager and then exported as C3D files. Kinematic parameters were quantified using Visual 3-D (C-Motion Inc., Gaithersburg, MD, USA). As all participants were right foot dominant, values were extracted from this side, and symmetry was assumed [12], the bilateral side was utilised only in the calculation of peak power at the centre of mass for which the total vertical GRF applied to the body was required. Marker data was smoothed using a low-pass Butterworth 4th order zero-lag filter, at a cut off frequency of 6 Hz [19]. Kinematics of the hip, knee, ankle and trunk were quantified using an XYZ cardan sequence of rotations, and joint moments using Newton–Euler inverse dynamics [18]. All data were normalised to 100% of the squat via the first and second instances of maximal hip flexion [20]. A further time point at the mid-point of the lift that separated the descent and ascent phases was identified using the lowest position of the bar [21].

Three-dimensional kinematic measures from the hip, knee and ankle, which were extracted for statistical analysis, were (1) peak angle and (2) angular range of motion (ROM) from initiation to peak angle. In addition, sagittal plane measures from the trunk of (1) peak flexion and (2) angular range of motion (ROM) were extracted. Joint power in the sagittal plane of the hip knee and ankle joints was calculated using the joint power function within Visual 3D. In accordance with Stone et al. [22], the integral of the power at each joint during the ascent phase was calculated using a trapezoidal function, to quantify energy production at each joint. The percentage (%) joint power contribution relative to total power was calculated as the quotient of energy production from each joint (described above) and the sum of the total energy production from the three joints [22].

The total lift duration was also calculated using the time difference from the initiation to the end of each repetition, and the absolute duration of the ascent/descent phases (s) was also extracted as was the percentage (%) duration of the ascent/descent phases, expressed as a function of the total lift duration. In addition, the maximum vertical velocity (m/s) and acceleration (m/s^2^) of the barbell during the ascent phase was quantified. The maximum extent to which the knee joint centre translated anteriorly and laterally during the squat movement was also calculated using Visual 3D. The net distances were normalised to the length of the shank segment and expressed as a percentage (%) [5].

Quadriceps force was estimated using a musculoskeletal model. The quadriceps force was resolved by dividing the external knee flexor moment from inverse-dynamics by the moment arm of the quadriceps muscle [23]. The moment arm of the quadriceps was calculated by fitting a 2nd order polynomial curve to the knee flexion angle-quadriceps moment arm data presented by van Eijden et al. [23].

Hamstring, gluteus maximus, soleus and gastrocnemius forces were also estimated using musculoskeletal modelling approaches [24]. The hamstring and gluteus maximus forces were calculated firstly using the hip extensor moment from inverse dynamics and the hamstrings and gluteus maximus cross-sectional areas, which determined the extent of the joint moment attributable to each muscle [25]. The hamstring muscle forces were then calculated by dividing the hip extensor moment attributable to each muscle by the muscle moment arms [26]. The moment arms were obtained by fitting a 2nd order polynomial curve to the hip flexion angle-hamstrings/gluteus maximus moment arm data of Nemeth and Ohlsen, [26]. In addition, the gastrocnemius and soleus forces were calculated firstly by quantifying the ankle plantarflexor force, which was resolved by dividing the dorsiflexion moment from inverse dynamics by the Achilles tendon moment arm. The Achilles tendon moment arm was calculated by fitting a 2nd order polynomial curve to the dorsiflexion angle-Achilles tendon moment arm data of Self and Paine [27]. Plantarflexion force accredited to the gastrocnemius and soleus muscles was calculated via the cross-sectional area of this muscle relative to the total volume of the triceps-surae [25].

All muscle forces were normalised by dividing the net values by body mass (N/kg). From the above processing, peak quadriceps, hamstring, gluteus maximus, soleus and gastrocnemius forces, as well as the forces at mid-lift, were extracted for statistical analysis. In addition, the impulse of these forces (N/kg·s) were calculated during the ascent and descent phases using a trapezoidal function. Finally, the peak rate of force development (PRFD) at each of the quadriceps, hamstring, gluteus maximus, soleus and gastrocnemius muscles, during the ascent phase was also extracted by obtaining the peak increase in muscle force between adjacent data points using the first derivative function within Visual 3D (N/kg/s).

Furthermore, patellar tendon force was quantified using a model adapted from Janssen et al. [28]. The knee flexion moment quantified using inverse dynamics was divided by the moment arm of the patellar tendon. The tendon moment arm was quantified by fitting a 2nd order polynomial curve to the knee flexion angle-patellar tendon moment arm data provided by Herzog and Read, [29]. The patellofemoral reaction force was calculated by multiplying the quadriceps force (described above) by a constant, which was obtained via Equation (1) below, using the data of van Eijden et al. [23]. Patellofemoral stress was also quantified by dividing the patellofemoral joint reaction force by the patellofemoral contact area. Patellofemoral contact areas were obtained by fitting a 2nd order polynomial curve to the sex-specific knee flexion angle-patellofemoral contact area data of Besier et al. [30].

**constant = (0.462 + 0.00147 ∗ knee flexion angle^2^ − 0.0000384 ∗ knee flexion angle^2^)/(1 − 0.0162 ∗ knee flexion angle + 0.000155 ∗ knee flexion angle^2^ − 0.000000698 ∗ knee flexion angle^3^)** (1)

The patellar tendon force (N/kg), patellofemoral force (N/kg) and patellofemoral stress (KPa/kg) as well as the values for the aforementioned indices at mid-lift were extracted following normalisation to body mass. The peak loading rate of the aforementioned knee force (N/kg/s) and stress (KPa/kg/s) parameters was calculated by obtaining the peak increase force/stress between adjacent data points during the squat, using the first derivative function within Visual 3D. In addition, the impulse of the aforementioned parameters (N/kg·s and KPa/kg·s) were calculated during the entire squat movement using a trapezoidal function.

From the force plate, peak vertical GRF (N/kg) during the ascent phase of the lift was extracted. The PRFD of the vertical GRF (N/kg/s) was also calculated by obtaining the peak increase in vertical GRF force between adjacent data points again using the first derivative function within Visual 3D. In addition, the impulse of the vertical and medial-lateral GRF’s (N/kg·s) were calculated during both the ascent and descent phases of the lift, again using a trapezoidal function. Furthermore, the peak power applied to the centre of mass (W/kg) during the ascent phase was extracted using a product of the vertical GRF and the vertical velocity of the three-dimensional kinematic model centre of mass within Visual 3D. In accordance with Lahti et al. [9], the angle of the resultant GRF vector relative to the horizontal plane was quantified at the instance of mid-lift by taking the product of an inverse tangent function and the quotient of the medial-lateral and vertical GRF’s.

Furthermore, to explore the effects of different foot placement angles on the location of the origin of the GRF, the position of the centre of pressure (COP) was first quantified relative to that of the foot centre of mass (mm) in both anterior-posterior and medial-lateral directions at the instance of mid-lift. Furthermore, in accordance with the recommendations of Sinclair et al. [18], the position of the COP relative to the model centre of mass (mm) in the medial-lateral direction was also extracted. Positive values in the anterior-posterior direction were indicative of COP being anterior and in the medial–lateral direction a positive value denotes that the COP is lateral to the foot/body centre of mass.

#### 2.2.5. Statistical Analyses

For comparative analyses, the foot placement angle was split according to the 33.3 and 66.6 percentiles allowing the creation of three separate groups: LOW (16.35 ± 3.62°, N = 23), MID (23.81 ± 1.71°, N = 24) and HIGH (31.58 ± 3.84°, N = 23). All experimental variables are presented as mean and standard deviations for each of the three foot-placement angle groups. Differences between the three groups were examined using between-participants linear mixed effects models with group modelled as a fixed factor and random intercepts by participants [31]. All analyses were conducted using SPSS v27 (IBM, SPSS, New York, NY, USA), and statistical significance accepted at the *p* ≤ 0.05 level.

### 2.3. Experiment 2

#### 2.3.1. Participants

An a priori sample size calculation for paired condition comparisons was undertaken to detect the same difference in peak power outlined in experiment 1, it was determined that in order to achieve α = 5% and β = 0.80 a total of 20 participants would be required. Male (age: 26.81 ± 4.45 years, stature: 176.17 ± 4.88 cm, mass: 77.17 ± 7.15 kg and 1RM back squat: 121.33 ± 16.72 kg) lifters took part in experiment 2. The same inclusion criteria as experiment 1 was adopted.

#### 2.3.2. Procedure

Kinetic and kinematic information was obtained using the procedure and biomechanical modelling approach outlined in experiment 1 and participants once again wore the same footwear.

#### 2.3.3. Squat Protocol

The same number of repetitions, loads and warm-up procedures as experiment 1 were adopted. To explore the effects of manipulating the foot-placement angle based on previous analyses [5], four experimental foot conditions were examined: 0°, 21°, and 42°, as well as participants own self-selected foot placement position henceforth named control, which had a measured foot placement angle of 19.56 ± 6.45°. To ensure the correct positioning of the feet, laminate paper was attached to the force plate marking each of the designated foot placement angles [5]. Participants performed in each of the four conditions in a counterbalanced order.

#### 2.3.4. Processing

The same processing techniques as experiment 1 were adopted and the same experimental variables were extracted.

#### 2.3.5. Statistical Analyses

Differences between the four foot-placement angle were examined using within participants linear mixed effects models with the foot placement condition modelled as a fixed factor and random intercepts by participants. The same statistical principles and reporting as experiment 1 were adhered to.

## 3. Results

### 3.1. Experiment 1

#### 3.1.1. Kinetic and Temporal Parameters

Kinetic and temporal parameters from experiment 1 are presented in Table 1. The angle of the GRF vector was significantly larger in the LOW group compared to HIGH. In addition, the medial GRF impulse in the ascent phase was significantly greater in the HIGH group compared to LOW and in the HIGH group compared to MID and LOW groups in the descent phase.

#### 3.1.2. Muscle Forces

Muscle force parameters from experiment 1 are presented in Table 2. No significant (*p* > 0.05) differences in muscle forces were observed.

#### 3.1.3. Three-Dimensional Kinematics

Three-dimensional kinematic parameters from experiment 1 are presented in Table 3. Coronal plane knee ROM was significantly greater in the LOW compared to the HIGH group.

#### 3.1.4. Joint Loads

Joint load parameters from experiment 1 are presented in Table 4. Peak patellofemoral stress was significantly greater in the HIGH compared to the MID and LOW groups.

### 3.2. Experiment 2

#### 3.2.1. Kinetic and Temporal Parameters

Kinetic and temporal parameters from experiment 2 are presented in Table 5. Bar velocity was significantly greater in the control condition compared to 0°. The angle of the GRF vector was significantly larger in the 0° condition compared to 21° and 42°, and also in the control and 21° conditions compared to 42°. The medial GRF impulse was significantly larger in both descent and ascent phases in the 21° and 42° conditions compared to 0°. In addition, the medial GRF impulse was significantly larger in both descent and ascent phases in the 42° compared to 21° and control conditions.

The anterior knee translation was significantly lower and the lateral knee translation significantly greater in the 21° and 42° conditions compared to 0°. In addition, anterior knee translation was significantly lower and lateral knee translation significantly greater in the 42° compared to 21° and control conditions. The COP was significantly more lateral to the foot centre of mass in the 21°, 42° and control conditions compared to 0° and also in the control condition compared to 42°. The COP was significantly more anterior to the foot centre of mass in the 0° compared to 42° and control conditions, and in the control condition compared to 42°. In addition, the COP was significantly more lateral to the body centre of mass in the 42° compared to the 0°, 21° and control conditions, and in the 21° condition compared to 0°. Percentage energy produced at the hip was significantly greater in the 0° compared to the control. The percentage energy produced at the knee was significantly greater in the control compared to the 0° conditions and the energy produced at the knee and ankle was significantly greater in the 0° condition compared to the control.

#### 3.2.2. Muscle Forces

Muscle force parameters from experiment 2 are presented in Table 6. Peak quadriceps force was significantly greater in the control condition compared to 0°, whereas gluteus maximus impulse during the descent phase was significantly greater in the 0° condition compared to control. In addition, gastrocnemius and soleus impulse during the ascent phase were significantly greater in the 0°, 21° and 42° conditions compared to control and gastrocnemius and soleus impulse during the descent phase were also significantly greater in the 0° condition compared to 42°. Finally, gastrocnemius and soleus forces at mid-lift were significantly greater in the 0°, 21° and control conditions compared to 42°

#### 3.2.3. Three-Dimensional Kinematics

Three-dimensional kinematic parameters from experiment 2 are presented in Table 7. Peak hip abduction and coronal plane hip ROM were significantly greater in the 42° condition compared to 0°, 21°and control conditions. Furthermore, peak abduction was significantly greater in the 21° condition compared to 0° and control. Peak hip internal rotation was significantly greater in the 0° compared to 21° and 42° conditions and in the control condition compared to 21°. Transverse plane hip ROM was also significantly greater in the 21°, 42° and control conditions compared to 0°.

Peak knee flexion and sagittal plane knee ROM were significantly greater in the 0°, 21° and control conditions compared to 42°. Peak knee internal rotation was significantly greater in the 0° condition compared to 21°, 42° and control, and also in the 21° and control conditions compared to 42°. Furthermore, knee internal rotation ROM was significantly larger in the 21° condition compared to 42°.

Peak ankle dorsiflexion was significantly greater in the 0° compared to the 21°, 42° and control conditions. In addition, peak dorsiflexion was significantly greater in the control condition compared to 21°and 42°. Peak ankle external rotation angle was significantly greater in the 42° compared to the and 0°, 21° and control conditions and in the 21°and control conditions compared to 0°. In addition, peak external rotation ROM was significantly greater in the 21° and 42° conditions compared to 0°.

#### 3.2.4. Joint Loads

Joint load parameters from experiment 2 are presented in Table 8. Peak patellofemoral force and stress were significantly greater in the control condition compared to 0°. In addition, the patellofemoral stress loading rate was significantly greater in the control condition compared to 21°. Peak patellar tendon force and force at mid-lift were significantly greater in the control compared to 0°, 21° and 42° conditions.

## 4. Discussion

The aim of the current investigation was to use a two-experiment approach to comparatively examine the effects of different habitual foot placement angles on kinetics, three-dimensional kinematics and muscle forces during the squat and also to explore the effects of manipulating the foot placement angle on the same biomechanical parameters. To the authors’ knowledge, this represents the first investigation to explore the aforementioned aims and may therefore provide further insight into the effects of different foot placement angles, which may be important for strength and conditioning coaches and sports therapists seeking to maximise training and rehabilitative adaptations.

Neither experiment saw any significant alterations in peak power as a function of the different experimental groups/conditions. However, the findings from experiments 1 and 2 showed that the angle of the GRF vector and the medial GRF impulse during both the ascent and descent phases of the squat were significantly influenced as a function of increases in the foot placement angle. Specifically, the GRF vector was greatest in the lowest and the medial GRF impulse greatest with larger foot placement angles. As the GRF vector initiates at the COP and orientates towards the centre of mass, it is proposed that this observation relates to the altered position of the COP relative to both the centre of mass of the body and also the foot in the larger foot placement angle conditions. Importantly, Lahti et al. [9] proposed that enhanced medially directed GRF’s during the squat may mediate a positive stimulus for athletes and coaches seeking to enhance proficiency in athletic disciplines requiring sprint performance and rapid changes of direction. Nagahara et al. [32] markedly showed that the impulse of the medial GRF was associated with enhanced sprint performance and played a key role in enhancing propulsive performance. Therefore, the current investigation suggests that owing to an increased ability to produce medially directed GRF’s linked to improved sprint and rapid change of direction performance, increased foot placement angles during the squat may be the most effective strategy for athletes seeking to enhance competence in these areas. Future randomised intervention studies are required before this can be substantiated and also to fully establish the effects of increasing medial GRF’s on other mechanical determinants of sprint performance.

Both experiment 1 and experiment 2 showed that knee joint loading was significantly influenced as a function of the experimental foot placement conditions. Experiment 1 revealed that patellofemoral joint stress was significantly greater in the HIGH group, whilst experiment 2 showed that both patellofemoral and patellar tendon loading indices were significantly greater in the control condition. Patellofemoral and patellar tendon loading are considered to be the primary biomechanical mechanisms linked to the initiation/progression of degenerative patellofemoral/patellar tendon pathologies [33,34]. From an injury prevention perspective, the current investigation first indicates that reducing the foot progression angle may attenuate the biomechanical mechanisms linked to the aetiology of knee pathologies, and from a rehabilitation perspective, active patients with patellofemoral/patellar tendon disorders may be encouraged by sports therapy professionals to adopt reduced foot progression angles as part of a graduated re-introduction of the squat into their exercise regimen.

Importantly, experiment 2 showed that muscle force parameters were significantly influenced by the experimental foot placement conditions. Specifically, the control condition mediated statistically increased quadriceps muscle forces in relation to 0°, whereas this condition was associated with significantly greater gluteus maximus, gastrocnemius and soleus forces compared to control. When combined with the findings in relation to joint energy production indices, this indicates that the control condition mediated a knee dominant squat technique, but the 0° produces a hip and ankle dominant strategy [22]. Skeletal muscle mechanical tension is the principal driver for hypertrophy [1], and the total muscle cross-sectional area is the key determiner of maximum muscle force production [35]. As training stimuli governs the magnitude of skeletal muscle adaptive responses [36]; this indicates that utilisation of self-selected foot placement angles may be advisable in athletes seeking to maximise quadriceps development, but that manipulation of the foot placement angle to 0° appears to be the most effective mechanism to promote gluteus maximus, gastrocnemius and soleus muscular development. Future longitudinal intervention analyses are required in order to explore the effects of different foot placement angles on indices of muscle hypertrophy using gold-standard magnetic resonance imaging or computed tomography approaches [37]. Furthermore, additional investigations may also be required to examine, during functional athletic movements, the longer-term efficacy of increasing recruitment of quadriceps at the expense of reductions in posterior chain muscle development.

In addition to the above, both experiment 1 and experiment 2 showed that lower extremity kinematics were significantly influenced by the experimental foot placement angle conditions. Specifically, it was revealed that coronal plane hip and knee kinematics were significantly greater in the condition with larger foot placement angles and that transverse plane hip and knee kinematics as well as ankle dorsiflexion indices were significantly larger in the 0° condition. This observation concurs with Lorenzetti et al. [5], and was likely mediated by the reductions in lateral knee displacement and corresponding increases in anterior knee displacement observed in the 0° foot placement condition.

A potential drawback to this study is that, across both experiments, only male recreational lifters were examined. Previous investigations have shown that squat biomechanics are significantly affected as a function of experience level and sex [5,18], so it is not known whether the same mechanical responses to the experimental conditions would have been mediated had a more experienced cohort, including female lifters, been examined. Therefore, it is proposed that the current study be repeated using a more experienced cohort of lifters of both sexes. Furthermore, a further potential limitation is that the current study adopted a musculoskeletal modelling-based approach for the quantification of muscle kinetics. Several mechanical assumptions are made in the creation of musculoskeletal models [38], which ultimately may influence the projected muscle kinetics. However, as in-vivo muscle force measures remain unfeasible due to the necessity of invasive testing procedures, the currently adopted approach represents the most feasible technique for the quantification of muscle forces at this time. A further drawback to the current study is that it represents an acute exploration of the effects of habitual and manipulated foot placement angles on squat biomechanics. Whilst the aims and methodologies from this investigation represent an extension of the current literature base as concurrent kinetic, three-dimensional kinematic and muscle force indices are examined, it remains unknown as to whether alterations in foot placement angle during the squat are able to mediate improvements in either skeletal muscle architecture or athletic performance indices. As such, a randomised controlled intervention investigation is clearly necessary in order to explore the effects of manipulating the foot placement angle of the squat on long-term physiological and pertinent performance-based outcomes in athletes.

## 5. Conclusions

In conclusion, the effects of foot placement angles on the biomechanics of the barbell back squat have received limited research attention. Therefore, the present study adds to the current scientific knowledge by providing a comprehensive two-experiment evaluation concerning the effects of foot placement angles on kinetics, kinematics and muscle forces during the squat. Importantly, across both experiments, increased foot placement angles led to significantly greater medial-lateral GRF impulse during the ascent and descent phases, yet reduced placement angles mediated reductions in patellofemoral/patellar tendon loading. In experiment 2, the 0° condition was found to mediate significant increases in gluteus maximus, gastrocnemius and soleus muscle kinetics, whereas the control condition significantly enhanced quadriceps forces. From a practical standpoint, the current investigation suggests owing to enhanced medially directed GRF’s, increased foot placement angles during the squat may be the most effective strategy for athletes seeking to enhance sprint and change of direction competence, whereas from an injury prevention perspective, reducing the foot progression angle may attenuate the biomechanical mechanisms linked to the aetiology of knee pathologies, and may also be adopted as part of a graduated re-introduction of the squat into a tailored rehabilitation regimen. Finally, a self-selected foot placement appears to be advisable in athletes seeking to maximise quadriceps hypertrophy, but an angle of 0° appears to be optimal in promoting posterior chain muscular development. So, whilst there does not appear to be an optimal foot placement angle, the findings from the current investigation provide additional insight into the effects of foot placement angle during the squat, that can be utilized effectively by both strength and conditioning and sports therapy practitioners seeking to maximize training and rehabilitative adaptations.

## Figures and Tables

**Table 1:** Kinetic and temporal parameters (mean  ±  SD) and statistical comparisons as a function of each experimental group.

	LOW		MID		HIGH		LOW vs. *MID*	*LOW* vs. *HIGH*	*MID* vs. *HIGH*
	Mean	*SD*	Mean	*SD*	Mean	*SD*	*p*-Value		
Peak power (W/kg)	12.73	3.88	12.55	2.97	13.71	3.93	0.859	0.411	0.263
Peak bar velocity (m/s)	0.97	0.18	0.95	0.13	1.00	0.21	0.606	0.631	0.311
Peak bar acceleration (m/s^2^)	4.17	1.50	4.82	1.72	4.92	2.25	0.181	0.202	0.869
Total duration (s)	2.42	0.42	2.23	0.33	2.31	0.47	0.100	0.449	0.482
Ascent duration (s)	1.18	0.22	1.11	0.16	1.13	0.20	0.190	0.415	0.675
Ascent percent duration (%)	49.12	4.43	49.95	4.71	49.20	3.73	0.544	0.949	0.556
Knee anterior translation (%)	43.53	12.01	48.10	9.39	48.15	7.55	0.156	0.134	0.983
Knee lateral translation (%)	24.48	9.28	22.21	5.49	26.58	9.08	0.313	0.458	0.054
Peak vertical force (N/kg)	12.53	2.93	13.15	2.59	13.69	2.45	0.668	0.114	0.133
PRFD (N/kg/s)	61.08	26.53	66.47	25.52	74.12	29.74	0.486	0.132	0.353
Medial GRF impulse ascent (N/kg·s)	1.37	0.42	1.54	0.53	1.87	0.70	0.238	0.007 *	0.081
Vertical GRF impulse ascent (N/kg·s)	10.54	2.84	10.24	2.02	10.95	2.81	0.682	0.631	0.328
Medial GRF impulse descent (N/kg·s)	1.39	0.44	1.36	0.61	1.76	0.65	0.810	0.035 *	0.036 *
Vertical GRF impulse descent (N/kg·s)	11.29	3.16	10.57	2.95	11.74	3.90	0.425	0.681	0.256
Squat depth (m)	0.47	0.08	0.47	0.09	0.49	0.09	0.896	0.429	0.499
GRF vector angle (°)	86.50	2.41	86.28	3.01	84.49	3.91	0.789	0.046 *	0.087
Medial-lateral COP position relative to foot COM (mm)	11.04	13.81	9.50	16.70	8.87	15.59	0.457	0.950	0.472
Anterior-posterior COP position relative to foot COM (mm)	−1.50	17.53	−2.93	49.75	−3.40	14.64	0.228	0.602	0.371
Medial-lateral COP position relative to body COM (mm)	238.91	27.94	243.11	27.08	260.33	41.45	0.608	0.048 *	0.100
Hip energy (%)	38.47	10.62	37.73	12.25	37.35	11.92	0.829	0.744	0.915
Knee energy (%)	53.15	9.93	54.00	10.86	54.14	10.55	0.782	0.749	0.966
Ankle energy (%)	8.39	3.27	8.27	3.11	8.51	3.28	0.899	0.899	0.795

Note: * + bold text denotes statistical significance.

**Table 2:** Muscle forces (mean  ±  SD) and statistical comparisons as a function of each experimental group.

	LOW		MID		HIGH		LOW vs. *MID*	*LOW* vs. *HIGH*	*MID* vs. *HIGH*
	Mean	*SD*	Mean	*SD*	Mean	*SD*	*p*-Value		
Peak quadriceps force (N/kg)	69.22	19.17	73.30	12.61	81.12	21.85	0.395	0.062	0.140
Quadriceps impulse ascent (N/kg s)	43.08	13.56	42.18	8.80	46.05	12.72	0.789	0.459	0.234
Quadriceps impulse descent (N/kg s)	49.74	17.90	47.08	13.73	54.52	17.09	0.572	0.370	0.109
Quadriceps PRFD (N/kg/s)	317.49	228.60	386.97	233.43	378.28	117.54	0.314	0.274	0.876
Quadriceps force at mid-lift (N/kg)	59.73	20.61	63.92	15.43	70.42	25.86	0.437	0.137	0.301
Peak gluteus maximus force (N/kg)	21.95	9.56	23.75	10.03	28.06	13.96	0.537	0.097	0.232
Gluteus maximus impulse ascent (N/kg·s)	10.29	4.39	9.99	3.65	11.04	3.05	0.802	0.510	0.295
Gluteus maximus impulse descent (N/kg·s)	9.84	4.04	9.66	3.75	10.37	3.60	0.877	0.646	0.515
Gluteus maximus PRFD (N/kg/s)	82.58	47.92	114.23	73.76	160.68	188.89	0.095	0.067	0.270
Gluteus maximus force at mid-lift (N/kg)	21.02	8.76	22.45	9.46	27.14	13.76	0.598	0.086	0.182
Peak hamstring force (N/kg)	44.89	28.89	48.66	25.03	48.54	25.68	0.637	0.660	0.987
Hamstring impulse ascent (N/kg·s)	20.12	10.86	19.48	8.02	20.49	10.15	0.819	0.910	0.710
Hamstring impulse descent (N/kg s)	19.70	10.19	18.61	7.46	19.42	9.53	0.680	0.926	0.749
Hamstring PRFD (N/kg/s)	160.68	104.87	229.53	173.95	238.33	226.21	0.115	0.152	0.883
Hamstring force at mid-lift (N/kg)	42.70	26.27	46.10	24.31	46.37	23.80	0.651	0.630	0.969
Peak gastrocnemius force (N/kg)	6.94	2.34	6.99	1.96	7.07	1.66	0.936	0.831	0.881
Gastrocnemius impulse ascent (N/kg·s)	5.30	2.37	4.99	1.84	5.15	1.40	0.625	0.803	0.745
Gastrocnemius impulse descent (N/kg·s)	4.62	1.80	4.43	2.09	4.25	1.60	0.752	0.473	0.736
Gastrocnemius PRFD (N/kg/s)	23.43	10.32	27.66	10.37	28.73	7.98	0.173	0.063	0.697
Gastrocnemius force at mid-lift (N/kg)	6.02	2.63	5.67	1.96	5.87	2.19	0.604	0.834	0.744
Peak soleus force (N/kg)	14.82	5.00	14.93	4.18	15.11	3.55	0.934	0.828	0.881
Soleus impulse ascent (N/kg·s)	11.30	5.05	10.59	4.09	10.99	2.98	0.599	0.807	0.704
Soleus impulse descent (N/kg·s)	9.78	3.88	9.41	4.52	9.00	3.43	0.770	0.484	0.731
Soleus PRFD (N/kg/s)	49.66	21.63	59.16	21.87	61.33	16.95	0.146	0.053	0.711
Soleus force at mid-lift (N/kg)	12.86	5.62	12.10	4.18	12.53	4.67	0.605	0.836	0.742

**Table 3:** Kinematic parameters (mean  ±  SD) and statistical comparisons as a function of each experimental group.

	LOW		MID		HIGH		LOW vs. *MID*	*LOW* vs. *HIGH*	*MID* vs. *HIGH*
	Mean	*SD*	Mean	*SD*	Mean	*SD*	*p*-Value		
	Trunk (sagittal plane + = flexion)								
Peak flexion (°)	29.74	8.45	30.76	8.01	29.21	10.08	0.676	0.851	0.564
ROM (°)	28.02	6.78	26.13	5.50	27.31	7.51	0.301	0.744	0.542
	Hip (sagittal plane + = flexion)								
Peak flexion (°)	97.73	22.68	100.23	30.51	95.44	24.50	0.756	0.749	0.562
ROM (°)	85.28	20.48	83.94	26.22	85.63	21.16	0.849	0.956	0.813
	Hip (coronal plane + = adduction)								
Peak abduction (°)	−29.09	7.53	−28.57	7.14	−27.91	9.62	0.809	0.653	0.794
ROM (°)	18.58	8.45	18.19	7.20	17.95	7.47	0.866	0.794	0.912
	Hip (transverse plane + = internal)								
Peak internal rotation (°)	6.23	16.82	8.49	9.75	8.28	8.77	0.577	0.616	0.940
ROM (°)	23.98	12.20	28.19	10.94	26.38	12.31	0.224	0.521	0.912
	Knee (sagittal plane + = flexion)								
Peak flexion (°)	120.68	14.26	117.85	12.78	121.60	10.44	0.482	0.807	0.284
ROM (°)	111.76	14.85	109.47	14.78	114.37	9.38	0.484	0.489	0.191
	Knee (coronal plane + = adduction)								
Peak adduction (°)	−1.25	9.86	−0.47	8.19	1.54	5.06	0.771	0.244	0.327
ROM (°)	9.18	6.80	6.21	5.18	3.91	2.72	0.101	0.002 *	0.069
	Knee (transverse plane + = internal)								
Peak internal rotation (°)	15.81	11.90	8.91	12.10	8.93	12.61	0.058	0.070	0.996
ROM (°)	18.49	10.61	13.11	12.29	13.88	6.17	0.120	0.085	0.791
	Ankle (sagittal plane transverse plane + = dorsiflexion)								
Peak dorsiflexion (°)	24.30	5.52	22.45	6.16	24.39	4.46	0.292	0.954	0.233
ROM (°)	24.74	4.11	24.60	5.99	25.55	5.04	0.925	0.566	0.567
	Ankle (coronal plane + = inversion)								
Peak eversion (°)	−7.67	5.44	-5.39	6.22	−5.30	7.10	0.193	0.220	0.965
ROM (°)	8.68	4.59	7.18	4.83	7.87	5.75	0.288	0.606	0.664
	Ankle (transverse plane + = internal rotation)								
Peak external rotation (°)	−0.52	5.22	−0.81	6.22	−1.30	5.09	0.865	0.617	0.771
ROM (°)	4.21	2.57	5.35	3.23	3.78	2.78	0.196	0.600	0.087

Note: * + bold text denotes statistical significance.

**Table 4:** Knee forces (mean  ±  SD) and statistical comparisons as a function of each experimental group.

	LOW		MID		HIGH		LOW vs. *MID*	*LOW* vs. *HIGH*	*MID* vs. *HIGH*
	Mean	*SD*	Mean	*SD*	Mean	*SD*	*p*-Value		
Peak patellar tendon force (N/kg)	60.92	24.15	58.25	17.88	64.91	18.46	0.670	0.542	0.221
Patellar tendon impulse (N/kg·s)	68.09	24.56	62.35	14.90	73.44	19.62	0.896	0.429	0.135
Patellar tendon force at mid-lift (N/kg)	55.69	23.25	55.11	18.48	62.87	18.30	0.926	0.261	0.160
Patellar tendon peak loading rate (N/kg/s)	253.61	230.31	289.79	200.23	262.78	90.47	0.884	0.922	0.756
Peak patellofemoral force (N/kg)	40.67	11.15	42.57	7.46	45.41	11.60	0.497	0.174	0.324
Patellofemoral impulse (N/kg·s)	53.07	16.81	50.78	12.08	57.96	15.01	0.595	0.314	0.079
Patellofemoral force at mid-lift (N/kg)	35.55	11.49	37.50	8.51	38.33	13.28	0.516	0.463	0.800
Patellofemoral peak loading rate (N/kg/s)	179.24	134.98	201.53	128.22	216.93	86.19	0.861	0.989	0.708
Peak patellofemoral stress (KPa/kg)	97.75	26.77	102.30	17.25	116.38	27.39	0.493	0.028 *	0.041 *
Patellofemoral stress impulse (KPa/kg·s)	148.83	48.39	138.21	31.39	159.88	46.05	0.378	0.442	0.067
Patellofemoral stress at mid-lift (KPa/kg)	72.71	25.98	78.92	20.88	85.99	33.00	0.374	0.146	0.386
Patellofemoral stress peak loading rate (KPa/kg/s)	591.58	324.34	631.63	286.27	774.02	302.87	0.867	0.912	0.760

Note: * + bold text denotes statistical significance.

**Table 5:** Kinetic and temporal parameters (mean  ±  SD) and statistical comparisons as a function of each experimental condition.

	0°		21°		42°		Control		0 vs. 21	0 vs. 42	0 vs. *Control*	21 vs. 42	21 vs. *Control*	42 vs. *Control*
	Mean	*SD*	Mean	*SD*	Mean	*SD*	Mean	*SD*	*p*-Value					
Peak power (W/kg)	13.68	4.75	13.58	4.59	13.20	4.57	13.60	4.44	0.838	0.325	0.878	0.123	0.968	0.379
Peak bar velocity (m/s)	0.92	0.16	0.92	0.13	0.92	0.13	0.95	0.19	0.383	0.896	p < 0.001 *	0.121	0.290	0.065
Peak bar acceleration (m/s^2^)	3.64	1.04	3.78	0.99	3.63	1.01	4.00	1.46	0.806	0.787	0.328	0.228	0.416	0.334
Total duration (s)	2.04	0.33	1.99	0.22	2.03	0.34	1.96	0.30	0.270	0.681	0.051	0.448	0.387	0.134
Ascent duration (s)	0.95	0.22	0.95	0.14	0.96	0.27	0.94	0.18	0.116	0.838	0.027*	0.491	0.486	0.209
Ascent percent duration (%)	47.22	4.56	48.03	3.65	47.95	6.43	48.24	4.13	0.025*	0.294	0.045*	0.927	0.677	0.665
Knee anterior translation (%)	50.19	9.80	48.19	9.85	43.15	9.25	48.95	10.03	p < 0.001*	p < 0.001 *	0.104	p < 0.001 *	0.092	p < 0.001 *
Knee lateral translation (%)	16.65	4.59	22.90	2.92	28.34	4.85	18.47	5.37	0.009 *	p < 0.001 *	0.057	p < 0.001 *	0.156	p < 0.001 *
Peak vertical force (N/kg)	9.52	2.61	9.61	2.66	9.46	2.64	9.77	2.60	0.516	0.448	0.661	0.164	0.305	0.039
PRFD (N/kg/s)	52.80	21.23	55.41	20.08	68.87	46.45	88.73	85.52	0.981	0.445	0.067	0.221	0.388	0.448
Medial GRF impulse ascent (N/kg·s)	0.95	0.37	1.04	0.39	1.25	0.49	0.98	0.38	0.009 *	p < 0.001 *	0.422	p < 0.001 *	0.162	p < 0.001 *
Vertical GRF impulse ascent (N/kg·s)	6.86	1.72	6.92	1.86	6.82	2.01	6.81	2.10	0.476	0.687	0.614	0.116	0.321	0.844
Medial GRF impulse descent (N/kg·s)	0.87	0.36	0.94	0.38	1.13	0.42	0.95	0.39	0.018 *	p < 0.001 *	0.163	p < 0.001 *	0.890	0.005 *
Vertical GRF impulse descent (N/kg·s)	8.02	2.69	7.74	2.52	7.85	2.93	7.71	2.67	0.160	0.377	0.188	0.720	0.891	0.618
Squat depth (m)	0.36	0.07	0.37	0.07	0.36	0.07	0.38	0.07	0.534	0.869	0.246	0.173	0.073	0.076
GRF vector angle (°)	85.83	4.20	84.94	3.52	84.13	3.91	85.27	3.46	0.009 *	p < 0.001 *	0.188	p < 0.001 *	0.373	0.018 *
Medial-lateral COP position relative to foot COM (mm)	0.01	0.01	10.37	8.06	2.54	12.13	7.54	7.82	p < 0.001 *	p < 0.001 *	p < 0.001 *	0.115	0.089	p < 0.001 *
Anterior-posterior COP position relative to foot COM (mm)	7.25	20.37	−0.28	14.41	−5.47	10.10	−0.04	19.95	0.259	p < 0.001 *	0.033 *	0.203	0.438	0.024 *
Medial-lateral COP position relative to body COM (mm)	235.93	24.28	252.84	23.33	280.87	26.28	245.42	25.35	p < 0.001 *	p < 0.001 *	0.068	p < 0.001 *	0.195	p < 0.001 *
Hip energy (%)	34.24	7.81	33.53	8.01	32.29	9.71	32.16	8.42	0.313	0.208	0.001 *	0.281	0.243	0.927
Knee energy (%)	54.33	6.64	55.55	7.31	57.36	8.85	58.37	8.01	0.216	0.117	p < 0.001 *	0.056	0.271	0.408
Ankle energy (%)	11.43	3.84	10.91	3.37	10.35	2.95	9.47	2.81	0.557	0.172	0.003 *	0.431	0.111	0.151

Note: * +bold text denotes statistical significance.

**Table 6:** Muscle forces (mean  ±  SD) and statistical comparisons as a function of each experimental condition.

	0°		21°		42°		Control		0 vs. 21	0 vs. 42	0 vs. *Control*	21 vs. 42	21 vs. *Control*	42 vs. *Control*
	Mean	*SD*	Mean	*SD*	Mean	*SD*	Mean	*SD*	*p*-Value					
Peak quadriceps force (N/kg)	58.42	16.11	58.71	16.25	58.25	14.57	59.35	16.12	0.684	0.840	0.014 *	0.330	0.285	0.148
Quadriceps impulse ascent (N/kg.s)	29.33	8.24	29.70	8.68	30.33	8.86	29.95	9.29	0.429	0.093	0.315	0.284	0.702	0.583
Quadriceps impulse descent (N/kg.s)	36.16	13.10	34.28	12.37	33.85	11.67	34.56	12.43	0.072	0.130	0.223	0.655	0.799	0.489
Quadriceps PRFD (N/kg/s)	272.72	83.16	270.30	66.99	350.00	216.38	293.17	83.64	0.653	0.136	0.143	0.121	0.063	0.311
Quadriceps force at mid-lift (N/kg)	54.19	16.88	53.53	16.42	54.09	15.89	55.11	15.77	0.520	0.917	0.172	0.514	0.070	0.261
Peak gluteus maximus force (N/kg)	14.30	5.41	14.28	5.10	13.59	6.04	14.99	5.55	0.928	0.322	0.165	0.326	0.243	0.197
Gluteus maximus impulse ascent (N/kg·s)	6.73	2.75	7.41	3.42	6.40	3.51	6.53	2.94	0.234	0.561	0.653	0.324	0.354	0.746
Gluteus maximus impulse descent (N/kg·s)	7.41	3.42	7.23	3.15	7.22	4.36	6.99	3.16	0.343	0.751	0.016 *	0.989	0.092	0.694
Gluteus maximus PRFD (N/kg/s)	53.86	21.44	55.41	20.08	68.87	46.45	88.73	85.52	0.560	0.165	0.088	0.160	0.093	0.201
Gluteus maximus force at mid-lift (N/kg)	13.61	5.57	13.58	5.21	12.90	6.03	14.22	5.50	0.923	0.255	0.153	0.264	0.227	0.171
Peak hamstring force (N/kg)	35.19	14.57	35.02	13.86	32.37	16.24	36.90	15.55	0.802	0.220	0.200	0.237	0.270	0.170
Hamstring impulse ascent (N/kg·s)	15.08	6.48	15.34	6.57	14.28	8.41	14.93	7.20	0.332	0.421	0.713	0.280	0.496	0.584
Hamstring impulse descent (N/kg.s)	17.01	8.08	16.46	7.50	16.00	10.55	16.03	7.67	0.177	0.529	0.183	0.760	0.255	0.989
Hamstring PRFD (N/kg/s)	121.87	45.86	119.37	36.21	143.06	91.93	196.88	194.84	0.621	0.343	0.107	0.239	0.089	0.152
Hamstring force at mid-lift (N/kg)	33.53	14.84	33.32	13.96	30.78	16.22	35.02	15.24	0.761	0.179	0.193	0.202	0.245	0.154
Peak gastrocnemius force (N/kg)	5.05	2.26	5.05	2.12	4.78	1.88	5.01	1.93	0.996	0.206	0.882	0.093	0.831	0.053
Gastrocnemius impulse ascent (N/kg·s)	3.41	1.61	3.38	1.62	3.21	1.57	2.99	1.52	0.883	0.306	0.009 *	0.150	0.016 *	0.036 *
Gastrocnemius impulse descent (N/kg·s)	3.33	1.58	3.01	1.53	2.87	1.33	3.12	1.60	0.081	0.016 *	0.107	0.260	0.323	0.063
Gastrocnemius PRFD (N/kg/s)	15.75	5.98	19.41	8.12	18.69	6.61	19.16	6.87	0.160	0.147	0.158	0.470	0.837	0.673
Gastrocnemius force at mid-lift (N/kg)	4.30	2.16	4.00	1.90	3.42	1.61	4.11	2.10	0.359	p < 0.001 *	0.426	0.007*	0.644	0.002 *
Peak soleus force (N/kg)	10.77	4.82	10.78	4.52	10.21	4.01	10.70	4.13	0.996	0.206	0.882	0.143	0.831	0.053
Soleus impulse ascent (N/kg·s)	7.29	3.44	7.22	3.46	6.86	3.35	6.38	3.24	0.883	0.306	0.010 *	0.150	0.015 *	0.035 *
Soleus impulse descent (N/kg·s)	7.10	3.37	6.42	3.26	6.13	2.85	6.66	3.41	0.081	0.017 *	0.107	0.260	0.323	0.063
Soleus PRFD (N/kg/s)	33.62	12.78	41.43	17.33	39.90	14.10	40.90	14.67	0.136	0.143	0.152	0.470	0.837	0.673
Soleus force at mid-lift (N/kg)	9.18	4.61	8.54	4.06	7.30	3.43	8.77	4.48	0.359	***p < 0.001***	0.426	0.006 *	0.644	0.003 *

Note: * +bold text denotes statistical significance.

**Table 7:** Kinematic parameters (mean  ±  SD) and statistical comparisons as a function of each experimental condition.

	0°		21°		42°		Control		0 vs. 21	0 vs. 42	0 vs. *Control*	21 vs. 42	21 vs. *Control*	42 vs. *Control*
	Mean	*SD*	Mean	*SD*	Mean	*SD*	Mean	*SD*	*p*-Value					
	Trunk (sagittal plane + = flexion)													
Peak flexion (°)	32.52	6.52	32.47	5.88	30.96	6.17	32.25	6.65	0.946	0.063	0.667	0.017	0.707	0.115
ROM (°)	23.31	7.02	22.25	6.86	21.72	6.87	23.22	7.01	0.155	0.018	0.870	0.255	0.080	0.020
	Hip (sagittal plane + = flexion)													
Peak flexion (°)	81.80	20.55	81.25	21.38	80.93	20.37	81.32	21.62	0.242	0.074	0.322	0.087	0.926	0.091
ROM (°)	64.31	15.57	63.61	16.71	64.95	20.60	64.55	15.60	0.217	0.247	0.749	0.061	0.272	0.053
	Hip (coronal plane + = adduction)													
Peak abduction (°)	−19.66	6.16	−25.20	8.00	−33.37	13.34	−21.69	6.33	p < 0.001 *	p < 0.001 *	0.118	p < 0.001 *	0.001 *	p < 0.001 *
ROM (°)	11.82	6.49	15.79	7.72	21.68	13.33	12.64	5.96	p < 0.001 *	p < 0.001 *	0.239	0.002 *	0.001 *	p < 0.001 *
	Hip (transverse plane + = internal)													
Peak internal rotation (°)	7.28	10.47	5.00	11.62	−3.23	16.53	7.53	10.36	0.003 *	0.015 *	0.775	0.051	***0.002***	***0.021***
ROM (°)	16.60	9.22	23.31	9.49	25.45	8.24	20.58	10.86	p < 0.001 *	0.009 *	p < 0.001 *	0.455	0.142	0.168
	Knee (sagittal plane + = flexion)													
Peak flexion (°)	113.41	10.88	114.05	12.33	110.34	10.97	114.62	12.62	0.448	p < 0.001 *	0.246	0.002 *	0.415	p < 0.001 *
ROM (°)	101.68	13.03	101.54	13.19	98.26	13.48	102.39	15.17	0.852	0.003 *	0.532	0.003 *	0.310	0.013 *
	Knee (coronal plane + = adduction)													
Peak adduction (°)	0.78	6.52	2.18	6.38	2.08	7.73	1.01	5.92	0.111	0.154	0.550	0.917	0.099	0.293
ROM (°)	5.53	4.18	6.08	4.22	6.44	3.63	5.56	3.90	0.152	0.150	0.927	0.634	0.094	0.188
	Knee (transverse plane + = internal)													
Peak internal rotation (°)	12.44	12.58	9.28	13.18	4.94	12.04	10.56	12.57	p < 0.001 *	p < 0.001 *	p < 0.001 *	p < 0.001 *	0.070	p < 0.001 *
ROM (°)	13.56	12.44	14.34	10.30	11.65	9.63	12.72	10.77	0.388	0.105	0.241	p < 0.001 *	0.112	0.393
	Ankle (sagittal plane transverse plane + = dorsiflexion)													
Peak dorsiflexion (°)	30.44	5.83	28.60	6.56	24.18	5.38	29.75	5.31	0.006 *	p < 0.001 *	0.224	p < 0.001 *	***0.027***	p < 0.001 *
ROM (°)	29.19	5.46	29.25	6.02	28.12	5.04	28.86	5.97	0.886	0.231	0.346	0.139	0.160	0.122
	Ankle (coronal plane + = inversion)													
Peak eversion (°)	−4.81	5.65	−5.47	5.02	−3.70	3.78	−4.73	5.82	0.105	0.080	0.885	0.099	0.182	0.184
ROM (°)	6.81	4.83	6.61	4.68	5.49	3.64	6.85	4.92	0.532	0.111	0.887	0.228	0.522	0.136
	Ankle (transverse plane + = internal)													
Peak external rotation (°)	1.40	5.06	−4.34	4.07	−7.01	4.21	−1.05	4.17	p < 0.001 *	p < 0.001 *	p < 0.001 *	p < 0.001 *	p < 0.001 *	p < 0.001 *
ROM (°)	4.09	1.90	5.13	2.58	5.04	2.83	4.81	3.10	p < 0.001 *	0.009 *	0.058	0.741	0.255	0.449

Note: * + bold text denotes statistical significance.

**Table 8:** Knee forces (mean  ±  SD) and statistical comparisons as a function of each experimental condition.

	0°		21°		42°		Control		0 vs. 21	0 vs. 42	0 vs. *Control*	21 vs. 42	21 vs. *Control*	42 vs. *Control*
	Mean	*SD*	Mean	*SD*	Mean	*SD*	Mean	*SD*	*p*-Value					
Peak patellar tendon force (N/kg)	44.40	17.97	45.67	18.98	43.11	16.71	47.53	19.48	0.262	0.218	0.003 *	0.203	0.036 *	0.002 *
Patellar tendon impulse (N/kg·s)	46.85	16.92	46.09	16.68	44.82	15.64	47.24	17.96	0.412	0.059	0.713	0.152	0.307	0.064
Patellar tendon force at mid-lift (N/kg)	42.62	18.17	41.79	9.31	41.12	16.50	46.57	19.37	0.112	0.192	p < 0.001*	0.301	p < 0.001 *	0.001 *
Patellar tendon peak loading rate (N/kg/s)	189.11	75.02	199.76	56.23	282.86	256.98	212.72	79.09	0.236	0.136	0.011 *	0.150	0.230	0.264
Peak patellofemoral force (N/kg)	32.22	9.32	34.32	9.42	34.05	8.45	34.71	9.32	0.820	0.726	0.039 *	0.319	0.290	0.135
Patellofemoral impulse (N/kg·s)	37.41	11.53	36.48	11.58	36.65	11.15	36.69	11.95	0.235	0.340	0.422	0.826	0.804	0.964
Patellofemoral force at mid-lift (N/kg)	31.26	9.67	30.79	9.31	31.55	9.12	31.52	9.11	0.443	0.612	0.526	0.186	0.144	0.963
Patellofemoral peak loading rate (N/kg/s)	154.42	45.82	152.08	37.29	199.16	123.95	163.75	44.99	0.397	0.134	0.084	0.112	0.149	0.275
Peak patellofemoral stress (KPa/kg)	39.56	11.39	40.79	11.63	40.47	10.50	41.13	11.44	0.588	0.882	0.016 *	0.362	0.378	0.207
Patellofemoral stress impulse (KPa/kg·s)	47.17	14.17	46.20	14.74	46.67	13.98	46.72	15.34	0.312	0.585	0.703	0.591	0.657	0.966
Patellofemoral stress at mid-lift (KPa/kg)	35.81	11.89	35.23	11.66	36.50	11.15	36.03	11.53	0.433	0.274	0.666	0.088	0.206	0.552
Patellofemoral stress peak loading rate (KPa/kg/s)	207.51	68.92	206.71	52.31	252.96	135.00	226.85	71.28	0.890	0.161	0.037	0.150	0.039 *	0.447

Note: * + bold text denotes statistical significance.
